# Supplementary material for: Prevalence and determinants of anemia among pregnant women in East Africa; A multi-level analysis of recent Demographic and Health Surveys
Source: PLoS One. 2021 Apr 27;16(4):e0250560. doi: 10.1371/journal.pone.0250560 (PMC8078763; doi:10.1371/journal.pone.0250560)
Supplement: Attachment — Submitted filename: rebutal letter.docx [file pone.0250560.s001.docx]

**Rebuttal letter	Date march /08/2021**

**Subject; submission of revised manuscript (PONE-D-20-37697)**

### Prevalence and determinants of anemia among pregnant women in East Africa; a multilevel analysis of recent Demographic and Health Surveys

**Alemneh MekuriawLiyew**

**To PLOS ONE**

Dear all,

We would like to thank you for these constructive, building, and improvable comments on this manuscript that would improve the substance and content of the manuscript. We considered each comment and clarification questions of editors and reviewers on the manuscript thoroughly. Our point-by-point responses for each comment and question are described in detail on the following pages. Further, the details of changes were shown by track changes in the supplementary document attached. The manuscript language was checked by language professionals and we follow journal guidelines. I have attached recent comments in a point-by-point response.

**Version 1; editor’s comments**

1. Please ensure that your manuscript meets PLOS ONE's style requirements, including those for file naming.

**Authors’ response**; thank you dear editor we have prepared the documents based on PLOS ONE requirement.

2. We suggest you thoroughly copyedit your manuscript for language usage, spelling, and grammar. If you do not know anyone who can help you do this, you may wish to consider employing a professional scientific editing service.

**Authors’ response;** thanks, dear editor. We have corrected the grammatical and spelling errors in the revised manuscript.

3. Please reference and discuss the following relevant publication:

<https://www.ncbi.nlm.nih.gov/pmc/articles/PMC7485848/>

**Authors’ response; t**hank you dear editor for your commitment we have included the above reference. Please see the revised manuscript.

**Version 2; reviewers’ comments**

1. Is the manuscript technically sound, and do the data support the conclusions?
   The manuscript must describe a technically sound piece of scientific research with data that supports the conclusions. Experiments must have been conducted rigorously, with appropriate controls, replication, and sample sizes. The conclusions must be drawn appropriately based on the data presented.

Reviewer #1: Yes

Reviewer #2: Yes

**Authors’ response**; thank you, dear reviewers

2 Have the authors made all data underlying the findings in their manuscript fully available?
  The [PLOS Data policy](http://www.plosone.org/static/policies.action#sharing) requires authors to make all data underlying the findings described in their manuscript fully available without restriction, with rare exceptions (please refer to the Data Availability Statement in the manuscript PDF file). The data should be provided as part of the manuscript or its supporting information, or deposited to a public repository. For example, in addition to summary statistics, the data points behind means, medians and variance measures should be available. If there are restrictions on publicly sharing data—e.g. participant privacy or use of data from a third party—those must be specified.

Reviewer #1: Yes

Reviewer #2: Yes

**Authors’ response**; thanks dear reviewers

3. Is the manuscript presented in an intelligible fashion and written in Standard English?
   PLOS ONE does not copyedit accepted manuscripts, so the language in submitted articles must be clear, correct, and unambiguous. Any typographical or grammatical errors should be corrected at revision, so please note any specific errors here.

Reviewer #1: No

Reviewer #2: Yes

**Authors’ response**; thanks a lot dear reviewers we have critically improved the readability of the manuscript. Please see the revised version.

**Reviewer 1 Comments**

After had carefully read the manuscript and take notes on overall strengths and weaknesses, I share my comments below.
**General comments**
**comment 1;** this study has a considerable prevalence of anemia in pregnant women in East Africa. It also presents the determinants related to anemia in pregnant women. However, the results presented did not differ from other studies already published in this area, and some researches and analyses published in this journal are very similar to this article.
- There are some typos in the text that need to be carefully revised
- Broadly, it is not clear what this study adds to the broader literature on determinants of anemia among pregnant women.
- The authors need to provide a more robust justification or highlight why this study is necessary and adds more information to the previous studies.

**Authors’ response;** thanks a lot dear reviewer for your critical comment to improve the manuscript. We accepted your comment and included sound justification in the revised manuscript. Though there are several publications for anemia during pregnancy so far, none of them give a summary or pooled prevalence of anemia and related determinants in east Africa. All are either country-based or sub-country-based studies. Nowadays integrating east Africa in different aspects including health is encouraged. Therefore, the findings from this study could have an important role to design maternal health programs in East Africa in an integrated approach. We have included a well-elaborated justification with clear citations in the revised manuscript. Please see paragraph 6 lines 79-93 of the revised manuscript.
**Comment 2;** In the first paragraph, the authors introduce concepts about anemia and how it affects pregnancy. I recommend starting the introduction in the second paragraph to present the global problem of anemia and its prevalence in Southeast Asia and Africa. - Also, I suggest addressing more issues of how the article differs from articles already published on this topic (similar with women of reproductive age - <https://doi.org/10.1371/journal.pone.0238957>).

**Authors’ response**; thanks a lot dear reviewer effort to improve the manuscript. As per your comment, the second paragraph describes the global burden of anemia during pregnancy followed by South East Asia Africa, sub-Saharan Africa, and East African countries. We also justified the benefit of the current study over the above-given article. We kindly request to see the revised manuscript.
**Results**
**Comment 3**; the readers would benefit if they had described the analysis plan (a figure or written) and how the analysis levels were included based on the previous literature and the novelty of this paper. Also, be clearer how variables were chosen, as models control for covariates.

**Authors’ response;** thanks, dear reviewer. The analysis was not based on the traditional regression methods. We used the advanced model with the multilevel analysis approached. Therefore, as per your comment, we clearly described the type of model used (multilevel binary logistic regression) level one and level two variables.

**Reviewer #2 comments**

**Comment and suggestion;** The manuscript addresses a relevant topic for improving the nutritional quality of pregnant women in East Africa. However, corrections and clarity are necessary for some issues, especially regarding the description of the method.
I made some comments that I hope will help you improve the manuscript.
Despite I am not a native of English it is important to review the writing of your manuscript. I had some difficulty understanding some phrases around your text.

**Authors’ response;** thanks dear reviewer we accepted your comment and the readability of the revised manuscript is well improved.
**1) Introduction**
**comment 2;** References are not always adequately used nor updated. The authors cite 39 references. Only 12 are from the last 5 years (2016-2020). One-third of the references were published more than 10 years ago.

**Authors’ response;** thank your dear reviewer for your strong effort to improve the quality of our manuscript and to keep the scientific rigor. We tried to search for the most recent publications. While doing so we replaced some old references with the updated ones. However, we are forced to use some old references due to lack of the updated articles**.**
**2) Methods**
comment 3; The study uses data from the Demographic and Health Surveys (DHS), which are nationally representative surveys and comparable across countries. However, I would suggest including a more detailed description of the DHS and the variables used. This reference may be useful: Daniel J Corsi, Melissa Neuman, Jocelyn E Finlay, SV Subramanian, Demographic and health surveys: a profile, International Journal of Epidemiology, Volume 41, Issue 6, December 2012, Pages 1602–1613, <https://doi.org/10.1093/ije/dys184>
It is important to highlight whether the pregnant women selected from DHS for the present study constitute a representative sample at the national level.

**Authors’ response;** thank you dear reviewer we strongly appreciate your commitment. We learned a lot from **“**Daniel J Corsi, Melissa Neuman, Jocelyn E Finlay, SV Subramanian, Demographic and health surveys: a profile, International Journal of Epidemiology, Volume 41, Issue 6, December 2012, Pages 1602–1613, <https://doi.org/10.1093/ije/dys184>” article. Consequently, we provided a detailed description of DHS data. Please see the revised manuscript.
**Comment 4;** Dependent variable: the definition of anemia is confusing. Why does the classification of anemia severity refer to women of reproductive age? “severe (if Hgb value <7 g / dL), and moderate (if Hgb value 7–9.9 g / dL) in women of reproductive age and mild (if Hgb level is 10.0–10.9 g/dL) in pregnant women. For this study, we recategorized anemia level as anemic which is coded as ‘1’ and non-anemic which is coded as ‘0’ from the previous classifications (no, mild, moderate, and severe).” Why did '0' include: no, mild, moderate, and severe?

**Authors’ response;** thank you, dear reviewer. Sorry for creating confusion. It is the grammatical error in the sentence construction “0” which includes only nonanemic ones. All other categories such as mild, moderate and severe are coded as “1’’. Generally, we paraphrased the whole paragraph in this section to improve the clarity.

**Comment 5**; Independent variables: it is necessary to describe each variable in more detail.
The order of presentation of the variables is very confusing. I would suggest that the authors review the order of the presentation. Is it possible to present the broader social variables first? Example: 1) place of residence (urban, rural); 2) level of poverty (high, low); 3) level of illiteracy (high, low);4)community health insurance (high, low). AFTER presenting individual social, economic, and demographic variables: First, the broader social variables: 1-Wealth status (poorest, poor, middle, rich, richest); 2- Type of source of drinking water (improved, not improved); 3-type of toilet facility (improved, not improved); 4-Covered by health insurance (yes, no); 5-distance from the health facility (big problem, not a big problem); 5-media exposure (yes, no). I would suggest that the authors describe what these variables refer to, for example, media exposure… Then, present the most proximal individual variables: 1-age (describe the age groups: 15-19, 20-24, 25-29, 30-34, 35-39, >= 40); 2-maternal education (no formal, primary, secondary, higher); 3-marital status (unmarried/married); 4-maternal working (yes, no); 5-sex of household head (male, female).In the sequence, present the obstetric variables: 1-parity (….); 2-history of a terminated pregnancy (yes, no); 3-iron supplementation (yes, no).

**Authors’ response;** Thanks a lot dear reviewer for your interesting and constructive comments. We described each variable in detail by providing how it is categorized. Regarding the order of presentation of independent variables as you said, it is possible to present the variables from broader ones to most proximal ones in traditional regression analysis. However, in multi-level analysis, the conceptualization and presentation of variables should indicate the analytic levels. For example, in our case, we do have two level variables i.e individual level (level one) and community (country) level (level two). So in our method, we first presented all variables that operate at an individual level, and next, we presented level two variables. We also follow the same approach in the result section again. First, individual-level variables were described.

3) Results
**comment 6**; In Table 2, I would suggest presenting the variables in the same order as described in the methods. Besides, corrections are necessary: standardization of decimal places; in the wealth status variable, the middle is out of place; in the age group it needs to correct 15-29 years and 34 years old in two groups.
**Authors’ response;** thanks dear reviewer we appreciate your critics reading. We corrected all the issues accordingly**.**

**Comment 7**; I would suggest including the CIs of all countries in Figure 1, and highlighting the values.
The item “Random effect and model comparison” presents Tables 2, but wouldn't it be Table 3? In Table 3, I would suggest repeating the meaning of the acronyms in a footnote, and include the p-value.

**Authors’ response;** thanks a lot dear reviewer. Sorry for creating the inconvenience it is to say “table 3”. Now it is corrected acronyms were expanded at the footnote and the p-value was included as per your suggestion. The CIs were also included in fig 1.
**Comment 8**; In the item “Determinants of anemia among pregnant women”, the description of the results is confusing concerning the individual and community variables. I would suggest presenting the community variables first and then the individual ones, following the same order described in the method.

**Authors’ response;** thank you, dear reviewer. As we have described in the above response for “comment 5”. In the same way with that of the method section here also we first described individual-level variables and then the community-level ones.
**Comment 9;** In the Introduction, the authors indicate that most factors associated with anemia during pregnancy are linked to socioeconomic conditions.
However, in the discussion, the comments do not refer to the worse social status of illiterate and unmarried women. For example, the authors justify that "This may be due to the low level of use of health services during pregnancy by illiterate women. There is evidence that these illiterate women did not seek prenatal care (ANC) (60)". Subsequently, the authors point out "In addition, illiterate women may be economically unstable (62) and may fail to meet nutritional needs (foods rich in vitamin A, iron and folic acid, etc.) during pregnancy, which can result in anemia”. However, to maintain consistency, this should be the first justification, as they do not have access to health services because they are socially excluded.

**Authors’ response;** thanks, reviewer. We accepted your comment and corrected it accordingly. We kindly request you to see the revised version.
**Comment 10;** Here too, the references are not always used adequately and are not always update. Examples: end of 1sr paragraph: “Besides, the higher prevalence of anemia in the current study could be attributed to the recent resurgences of malaria in East Africa due to climate change (46)”. To support their statement, the authors cite a study of 2002, but they should cite more recent studies.

**Authors’ response;** thanks dear reviewer for your suggestion we replaced the above reference with relatives recent publication. But we face a challenge to get updated references for most of the articles. Consequently, we are forced to use the existing and available kinds of literature.

**Thanks, aot!!!**
