# Supplementary material for: Prevalence and determinants of anemia among pregnant women in East Africa; A multi-level analysis of recent Demographic and Health Surveys
Source: PLoS One. 2021 Apr 27;16(4):e0250560. doi: 10.1371/journal.pone.0250560 (PMC8078763; doi:10.1371/journal.pone.0250560)
Supplement: Attachment — Submitted filename: rebutal letter.docx [file pone.0250560.s002.docx]

**Rebuttal letter	Date march /27/2021**

**Subject; submission of revised manuscript (PONE-D-20-37697R1)**

### Prevalence and determinants of anemia among pregnant women in East Africa; a multilevel analysis of recent Demographic and Health Surveys

**Alemneh Mekuriaw Liyew**

**To PLOS ONE**

Dear all,

We would like to thank you for these constructive, building, and improvable comments on this manuscript that would improve the substance and content of the manuscript. We considered each comment and clarification questions of editors and reviewers on the manuscript thoroughly. Our point-by-point responses for each comment and question are described in detail on the following pages. Further, the details of changes were shown by track changes in the supplementary document attached. We have attached recent comments in a point-by-point response.

**Version 1; editor’s comments**

1. Please ensure that your manuscript meets PLOS ONE's style requirements, including those for file naming.

**Authors’ response**; thank you dear editor we have prepared the documents based on PLOS ONE requirement.

2. Please review your reference list to ensure that it is complete and correct. If you have cited papers that have been retracted, please include the rationale for doing so in the manuscript text, or remove these references and replace them with relevant current references. Any changes to the reference list should be mentioned in the rebuttal letter that accompanies your revised manuscript. If you need to cite a retracted article, indicate the article’s retracted status in the References list and also include a citation and full reference for the retraction notice.

**Authors’ response;** thanks dear editor we can confirm that our reference is complete and correcte. Retracted paper is not cited.

**Version 2; reviewer’s comments**

1. Is the manuscript technically sound, and do the data support the conclusions?
   The manuscript must describe a technically sound piece of scientific research with data that supports the conclusions. Experiments must have been conducted rigorously, with appropriate controls, replication, and sample sizes. The conclusions must be drawn appropriately based on the data presented.

Reviewer #1: Yes

Reviewer #2: Yes

**Authors’ response**; thank you, dear reviewers

2 Have the authors made all data underlying the findings in their manuscript fully available?
  The [PLOS Data policy](http://www.plosone.org/static/policies.action#sharing) requires authors to make all data underlying the findings described in their manuscript fully available without restriction, with rare exceptions (please refer to the Data Availability Statement in the manuscript PDF file). The data should be provided as part of the manuscript or its supporting information, or deposited to a public repository. For example, in addition to summary statistics, the data points behind means, medians and variance measures should be available. If there are restrictions on publicly sharing data e.g. participant privacy or use of data from a third party—those must be specified.

Reviewer #1: Yes

Reviewer #2: Yes

**Authors’ response**; thanks dear reviewer

**Reviewer Comments**

**Comment 1;** The authors reviewed almost all the items highlighted, but there is a major problem, which refers to changes in the prevalence of anemia, without justification. There was a small change in the overall prevalence of anemia, but a substantial change in the prevalence of anemia in each of the countries, as shown in Figure 1:
1st version:
The prevalence of anemia among pregnant women in eastern Africa was 42.50 (95% CI: 41.52, 44.10) with a large difference between specific countries which ranges from 22.08% in Burundi to 51.88% in Zimbabwe (Fig. 1).
Figure 1: Burundi (22.08), Ethiopia (32.45), Madagascar (35.20), Malawi (37.32), Mozambique (38.02), Rwanda (38.64), Tanzania (44.94) ), Uganda (45.48), Zambia (51.88), Zimbabwe (58.99)
2nd version:
The prevalence of anemia among pregnant women in easten Africa was 41.82 (95% CI: 40.78, 42.87) with a large difference between specific countries which range from 23.36% in Rwanda to 57.10% in Tanzania (Fig. 1).
Figure 1: Burundi (46.76), Ethiopia (28.01), Madagascar (37.44), Malawi (45.12), Mozambique (50.64), Rwanda (23.38), Tanzania (57.10) ), Uganda (38.20), Zambia (41.19), Zimbabwe (33.07)
Despite these substantial changes in prevalence, the other analyzes have not changed and I have found no justification for these changes in the authors' response.

**Authors’ response;** thanks dear reviewer for your critical comment. We ask a great apology for creating such inconvenance and unnecessary confussion. As you know in the large survey data like DHS (the one we used ) there is a great difference in the weighted and unweighted prevalence. The weighted prevalence provides better and valid eastimate than the unweighted one . Thus,it is the weighted prevalence that we are expected to report. (Guide to DHS Statistic 2018). However, in the first version the unweighted proportion was reported though the sampling weight was adjusted in the regression analysis.

Therefore, in the revised version the weighted prevalence was conducted for each country (that is why it is completely different from the first version) as well as for the overall prevalence by using the weighting variable which is already available in in the datasets. So, the updated prevalence (second version) is valid estimate than the previous one for two reasons

1. The sampling weight was applied to produce the actual and reliable estimate
2. The confidence interval was included for those weighted prevalences

Regarding the regression analysis, the sampling weight was already adjusted in stata in all models to produce reliable estimates. That is why the findings in the first version and in the second version for the regression outputs are similar. As you know prevalence (descriptive only; for which we missed the sampling weight in the previous version) and regression analysis ( for which we accounted the sampling weight in the first version) are conducted separately. In describing the frequency for each independent variables we have already reported both the unweighted and weighted frequencies with respective percentage in both versions for clarity.

So, the mistake is that we fail to justify such happening in our previous point by point response. We ask a great appology for this.
**comment 2.** In Table 2:
a) I suggest including a line identifying the variables at the Individual-level and another line indicating the variables at the Community-level.
b) I continue to suggest maintaining the same order as the variables presented in the Method.
c) It is necessary to correct the maternal age of 15-29 years group.

**Authors’ response**; thanks dear reviewer. We have fully accepted three of your comments and corrected accordingly.
**comment 3** ;In Table 3:
a) In the variable Maternal occupation, in Model II, it is necessary to correct 1.2 (0.93,1.11). Would it be 1.02?
b) In the variable Maternal age: In Model III, what was the reference? Should not the asterisk in the 20 to 24 age group be in the 15 to 19 age group?

**Authors’ response;** we really appreciate your critical reading ability. We have corrected both of your comments accordingly.

**Thanks, a lot!!!**
